# Evaluation of initial patient setup methods for breast cancer between surface-guided radiation therapy and laser alignment based on skin marking in the Halcyon system

**DOI:** 10.1186/s13014-023-02250-3

**Published:** 2023-04-04

**Authors:** Seonghee Kang, Hyeongmin Jin, Ji Hyun Chang, Bum‑Sup Jang, Kyung Hwan Shin, Chang Heon Choi, Jung-in Kim

**Affiliations:** 1 Department of Radiation Oncology, Seoul National University Hospital, 101, Daehak-ro, Jongno-gu, Seoul, Republic of Korea; 2 Department of Radiation Oncology, Seoul National University College of Medicine, Seoul, Republic of Korea; 3 Biomedical Research Institute, Seoul National University Hospital, Seoul, Republic of Korea; 4 Institute of Radiation Medicine, Medical Research Center, Seoul National University, Seoul, Republic of Korea; 5 Cancer Research Institute, Seoul National University College of Medicine, Seoul, Republic of Korea

**Keywords:** SGRT, Halcyon, Residual rotational error, Patient setup

## Abstract

**Background:**

This study was conducted to evaluate the efficiency and accuracy of the daily patient setup for breast cancer patients by applying surface-guided radiation therapy (SGRT) using the Halcyon system instead of conventional laser alignment based on the skin marking method.

**Methods and materials:**

We retrospectively investigated 228 treatment fractions using two different initial patient setup methods. The accuracy of the residual rotational error of the SGRT system was evaluated by using an in-house breast phantom. The residual translational error was analyzed using the couch position difference in the vertical, longitudinal, and lateral directions between the reference computed tomography and daily kilo-voltage cone beam computed tomography acquired from the record and verification system. The residual rotational error (pitch, yaw, and roll) was also calculated using an auto rigid registration between the two images based on Velocity. The total setup time, which combined the initial setup time and imaging time, was analyzed to evaluate the efficiency of the daily patient setup for SGRT.

**Results:**

The average residual rotational errors using the in-house fabricated breast phantom for pitch, roll, and yaw were 0.14°, 0.13°, and 0.29°, respectively. The average differences in the couch positions for laser alignment based on the skin marking method were 2.7 ± 1.6 mm, 2.0 ± 1.2 mm, and 2.1 ± 1.0 mm for the vertical, longitudinal, and lateral directions, respectively. For SGRT, the average differences in the couch positions were 1.9 ± 1.2 mm, 2.9 ± 2.1 mm, and 1.9 ± 0.7 mm for the vertical, longitudinal, and lateral directions, respectively. The rotational errors for pitch, yaw, and roll without the surface-guided radiation therapy approach were 0.32 ± 0.30°, 0.51 ± 0.24°, and 0.29 ± 0.22°, respectively. For SGRT, the rotational errors were 0.30 ± 0.22°, 0.51 ± 0.26°, and 0.19 ± 0.13°, respectively. The average total setup times considering both the initial setup time and imaging time were 314 s and 331 s, respectively, with and without SGRT.

**Conclusion:**

We demonstrated that using SGRT improves the accuracy and efficiency of initial patient setups in breast cancer patients using the Halcyon system, which has limitations in correcting the rotational offset.

## Background

In recent years, surface-guided radiation therapy (SGRT), which uses the three-dimensional (3D) surface of the patient in real-time using optical imaging, has been widely adopted as a patient setup and monitoring method [1–9]. Because the accuracy of radiation therapy is closely related to the patient setup, it is important to verify that between the simulation and treatment, the patient position is consistent [10]. SGRT provides real-time six degrees of freedom (6-DOF) surface information in the treatment room for a reference body contour acquired from treatment planning CT [1, 2, 5, 6]. The 6-DOF information without an additional imaging dose can be used to monitor the intra-fractional motion during treatment and provide the necessary correction information for the patient’s reference position. In particular, SGRT allows for more accurate positioning compared with conventional laser alignment based on skin marking (LAS) and could reduce the extent of daily imaging in some cases [4]. In addition, it is an effective method for reducing the overall setup time, which can minimize the time required for image registration [7]. LAS, which is routinely used as a reference for the initial patient setup for treatment as well as for simulation in our hospital, has the advantage of being a simple and non-invasive method. However, it is primarily based on the therapist’s perspective, thus making the initial patient setup method user-dependent and inaccurate. In some cases, it corrects according to the skin rather than the position of the entire body because of the elasticity of human skin [5, 6].

With an increasing number of patients undergoing hypo-fractionated whole breast radiotherapy (WBRT) or accelerated partial breast irradiation (APBI), the use of intensity-modulated radiation therapy (IMRT) or volumetric modulated arc therapy (VMAT), which can achieve highly conformal dose distributions, has increased [11, 12]. However, uncertainties related to the patient setup may lead to inaccuracies in dose delivery; in particular, because of the steep dose gradient, the efficacy of IMRT and VMAT can be limited and the patient outcomes for both local tumor control and normal tissue complications can be affected [13]. In particular, because breast tissue is highly deformable, reproducing the patient setup is often challenging in patients with breast cancer. Several studies have shown SGRT to be a useful method for improving the accuracy and efficiency of the patient setup compared to conventional tattoo/laser-based method with the potential to reduce the frequency of routine image guided radiation therapy [1–9, 14–22].

The Halcyon™ linear accelerator (LINAC) (Varian Medical System, Palo Alto, CA, USA) is a bore-enclosed LINAC that corrects the patient setup position in every fraction using kV or MV imaging systems. Unlike the C-type LINAC, the patient’s initial setup is performed in the virtual isocenter, which is approximately 58 cm away from the treatment isocenter in the longitudinal direction, and the patient is then moved into the bore of the treatment isocenter. After moving to the treatment isocenter, the patient’s position is corrected by moving the couch value in terms of the difference value obtained by image registration between the reference imaging and daily imaging. However, it is difficult to correct for rotational errors because the couch in the Halcyon device only allows for translational movement [14, 15]. If a rotational error occurs during the initial setup, the additional imaging dose and the overall setup time increase because the patient must be manually repositioned. Therefore, the initial patient setup in the virtual isocenter is critical since it affects the accuracy and efficiency of the overall treatment. In this study, the accuracy to measure the residual rotational error of the SGRT system was evaluated using in-house breast phantom. Also, unlike most previous studies comparing two group using different initial setup methods, CBCTs obtained from each patient using both initial setup methods were analyzed to evaluate the accuracy and efficiency of SGRT compared with LAS method using Halcyon system. [3–7, 14–16]

## Methods and materials

After receiving institutional review board approval, a retrospective study was conducted on 38 breast cancer patients treated with the Halcyon LINAC in our hospital. The treatment simulation was performed with a Brilliance CT Big Bore (Philips, Cleveland, OH, USA) using a slice thickness of 2.5 mm and a breast board was used for immobilization and to achieve reproducibility of the patient’s position. The Halcyon LINAC, which contains a 6 MV flattening filter free (FFF) and a dual-layer multi-leaf collimator (MLC), allows fast and accurate IMRT and VMAT based on a kV cone beam CT imaging (kV CBCT) system. Because it is necessary to move from the virtual isocenter to the treatment isocenter after the initial setup, 2D or 3D images should be acquired for every fraction to ensure that the positioning is accurate. Compared with the C-type LINAC, the patient setup for Halcyon Linac can be corrected with only translational error because the treatment couch has 3-DOF. The daily initial setup for each patient was performed by aligning skin markings with room laser for three fractions and by using SGRT for three fractions. In the LAS method, after marking the skin with ink at the reference isocenter during CT simulation, the patients were aligned to the marked locations and were manually shifted by the therapists. For the SGRT method, body surfaces were reconstructed using an imaging system that was used to match the body contours acquired from the reference CT image. After acquiring 6 CBCTs (3 CBCTs with LAS method, 3 CBCTs with SGRT) per patient, a total of 228 CBCTs were analyzed.

### SGRT implementation

**Fig. 1:**
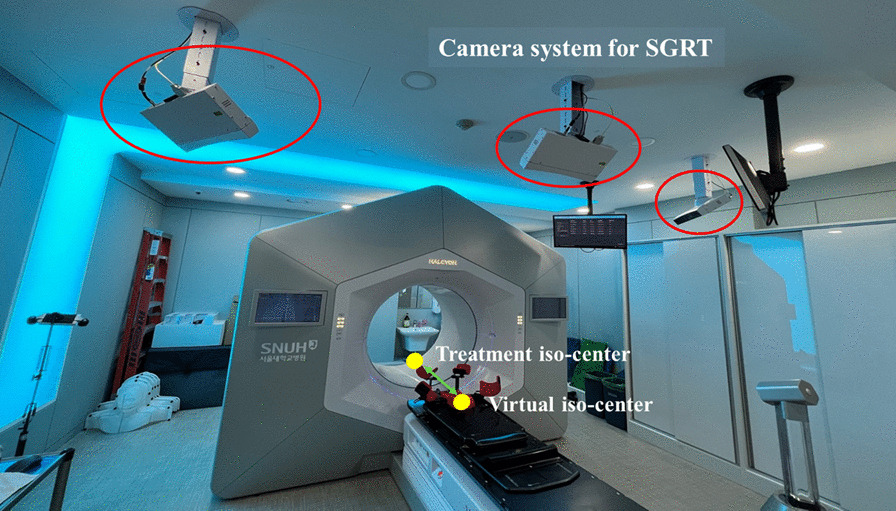
Patient setup monitoring system configured with the three-camera pod SGRT on the Halcyon system

In our study, the AlignRT image-guided system (Version 6.2, Vision RT Ltd., London, UK) was used for SGRT implementation. As shown in Fig. 1, AlignRT, consisting of a three-camera pod system, was installed on the ceiling and aligned to the virtual isocenter to monitor the patient’s initial setup out of the bore. System calibration was performed on the virtual isocenter owing to the area blocked by the O-ring type design. The daily QA for the SGRT system was performed using a plate with a circular pattern before treatment. When the maximum root mean square values representing the average discrepancy of the camera positions between the daily QA and system calibration exceeded 0.8 mm, the system was calibrated. While the initial setup of the patient was performed at the virtual isocenter, the reference body contour generated by the reference CT had the coordinates of the treatment isocenter; therefore, a dummy plan with a couch position without delta couch shift was generated using the Eclipse Scripting API. The region of interest (ROI) for breast cancer patients, which includes breast tissues, ribs, and the lateral aspect of the left and right breast to the midcoronal plane, was generated to monitor the difference in body contour quantitatively. The thresholds for the translational error (vertical, longitudinal, and lateral) and rotational error (pitch, roll, and yaw) were set to 3 mm and 3°, respectively. If the difference in the 6-DOF was within the thresholds, the couch was moved to the treatment isocenter for kV CBCT imaging.

### Accuracy verification for rotational error

**Fig. 2:**
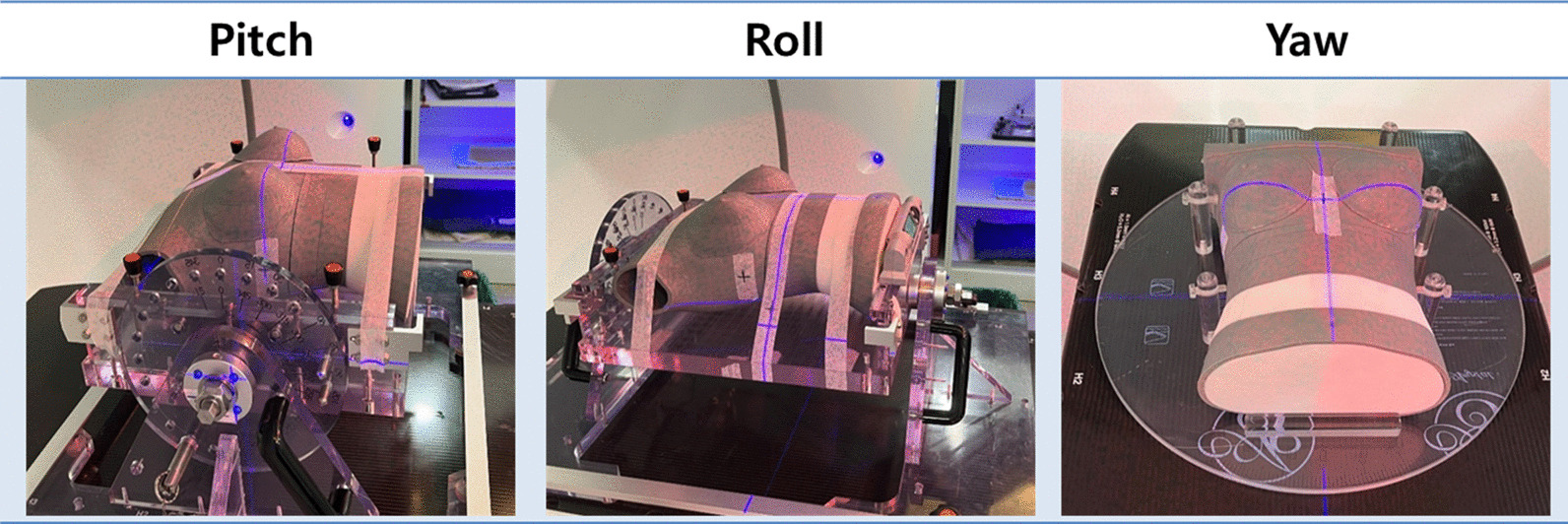
Phantom fabricated in-house on the base plate, which induced arbitrary rotational errors for evaluating the SGRT system

The accuracy of the residual rotational error of the SGRT system was evaluated by using an in-house breast phantom. An anthropomorphic phantom (Model 702 Adult ATOM Female, CIRS Inc., Norfolk, VA), which is sectional in design with traditional 25 mm thick sections, was scanned with a 1.5 mm slice thickness. A phantom designed with a thickness of 25 mm has dosimetric capabilities, but it is difficult to obtain reproducible surface imaging. Therefore, to be suitable for the SGRT system, a phantom with an opaque/matte, light-colored surface that reflects the projected light pattern was fabricated using 3D printing. The body contours were converted into the standard tessellation language format for 3D printing. The phantom was manufactured from amorphous thermoplastic material using a Zortrax M300 3D printer (Zortrax, Olsztyn, Poland). After CT scanning the 3D printed breast phantom, the body contour was used as reference in the same way as the patient cases. The phantom on the baseplate was set to a virtual isocenter, and rotational errors from − 1.5° to 1.5° in steps of 0.5°were induced for pitch, yaw, and roll using the baseplate, as shown in Fig. 2. The induced rotational errors were acquired using an SGRT system. Then, the rotational error was estimated by matching the body contour imported from Eclipse and acquired surface images using AlighRT, and the kV CBCT scans were acquired. The kV CBCTs were imported into Velocity™ (Version 4.1, Varian Medical Systems Inc.) to verify the residual rotational error using auto rigid registration between the kV CBCT and reference CT.

### Data acquisition and analysis

A total of 228 fractions including three fractions with LAS methods and three fractions with the SGRT for each patient acquired from thirty-eight patients prescribed dose of 43.2 Gy in 16 fractions were investigated. Twenty-nine of the patients underwent breast-conserving surgery and nine underwent mastectomy. The accuracy of the initial setup in terms of translational error was quantified by retrospectively analyzing the couch shifts between the reference CT and kV CBCT images. Because the Halcyon couch only moves the translational shift, both the kV CBCT and reference CT were exported into Velocity™ to compare the residual rotational error. The ROI was set to the appropriate dimensions in all three planes (axial, sagittal, and coronal) to include the chest wall and ribs to be fused. After fusing the two images, the rotational errors for the pitch, roll, and yaw were obtained from the automatic registration results. The setup efficiency was analyzed by comparing the initial setup and imaging times. The initial setup times were acquired by measuring the timestamp of the record and verification (R&V) system, starting from the instant the patient was loaded into the system and ending when the images were acquired. The imaging time was defined as from start of kV beam irradiation until after couch shift performed by the auto-and manual image registration by measuring the timestamps of R&V system. Patient data were analyzed by calculating mean, median, maximum, and quartile distributions. A paired t-test was used to establish the statistical significance of the results at *P* < 0.05.

## Results

### Accuracy verification for rotational error

**Fig. 3:**
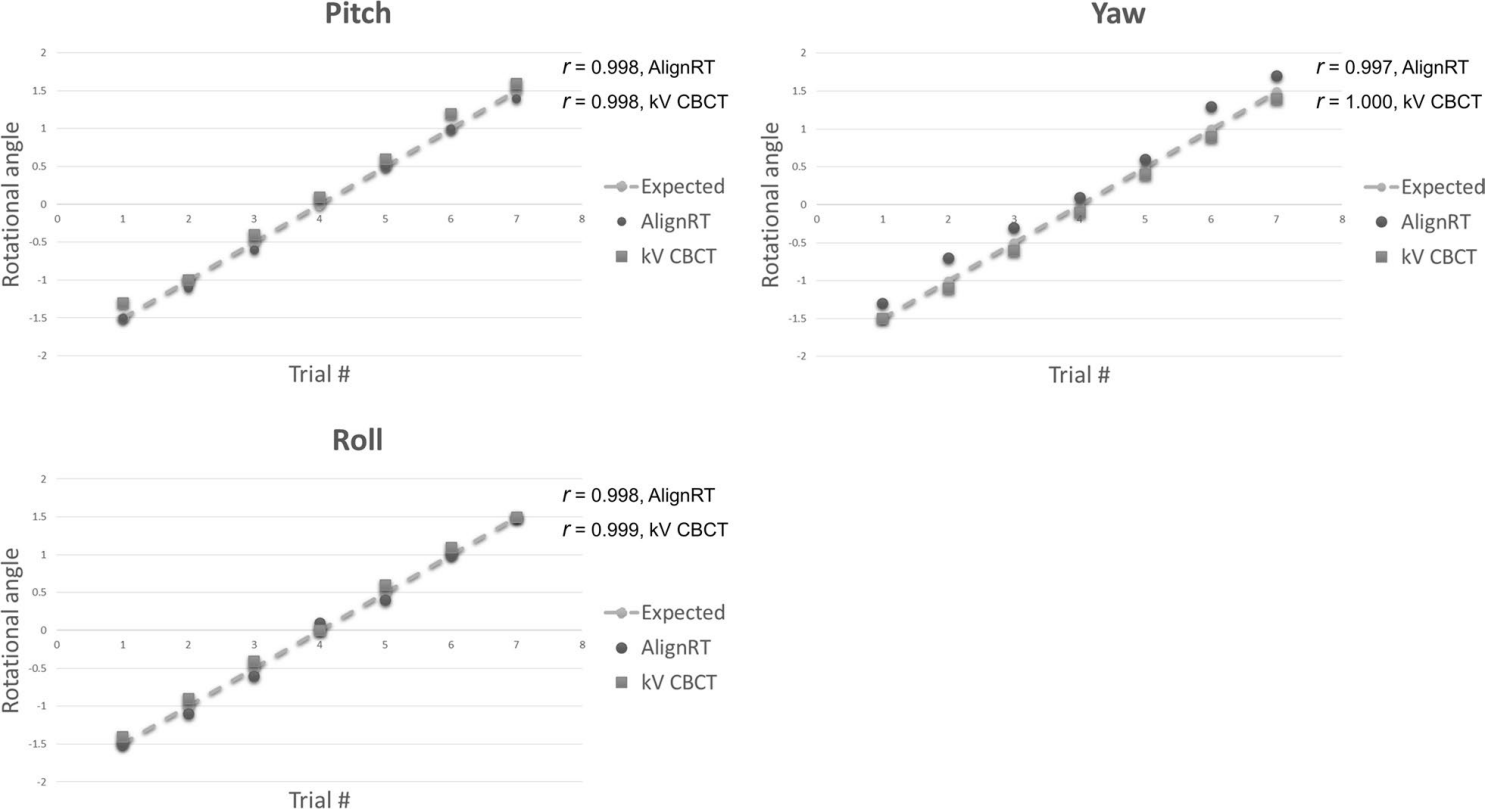
Residual rotational error acquired by inducing phantom rotation from − 1.5° to 1.5° in 0.5° increments

Figure 3 presents the results for the residual rotational error of the SGRT using an in-house phantom. These rotational errors indicate good agreement between SGRT and induced rotational error, compared with the rigid registration of the daily kV CBCT and reference CT. The mean residual rotational errors in the SGRT for pitch, yaw, and roll were 0.14°, 0.29°, and 0.13°, respectively. The largest error between the two systems was for yaw and the smallest difference was for roll. The maximum difference between the induced and SGRT systems in all directions was 0.40°.

### Initial setup accuracy

A comparison of the kV CBCT translational and rotational errors with and without SGRT is presented in Table 1. The average translational errors for the vertical, longitudinal, and lateral directions in the case of the LAS methods were 0.27 mm, 0.2 mm, and 0.21 mm, respectively. Compared with the LAS method, the average translational error using SGRT showed vertical and lateral improvements in the patient setup. However, it was confirmed that the translational error significantly decreased longitudinally using the LAS method. The average rotational errors for pitch, yaw, and roll using the LAS method were 0.32°, 0.51°, and 0.29°, respectively. By contrast, the average rotational difference for roll was significantly reduced using SGRT; however, yaw was similar to that of the LAS method. The respective average rotational errors for pitch, roll, and yaw using the SGRT were 0.3, 0.51, and 0.19.

**Table 1:** Comparison of translational and rotational errors acquired by the kV CBCT and the reference CT with and without SGRT

	Translational error (cm)			Rotational error (°)		
	Vertical	Longitudinal	Lateral	Pitch	Yaw	Roll
LAS						
Min	0.03	0.02	0.07	0.04	0.12	0.04
Median	0.28	0.19	0.18	0.18	0.45	0.26
Quartile 1	0.15	0.12	0.13	0.1	0.31	0.14
Quartile 3	0.36	0.25	0.25	0.45	0.74	0.34
Max	0.74	0.61	0.52	1.24	1.04	1.09
Mean	0.27	0.2	0.21	0.32	0.51	0.29
SGRT						
Min	0.02	0.05	0.06	0.03	0.06	0.02
Median	0.15	0.2	0.18	0.25	0.45	0.19
Quartile 1	0.12	0.15	0.15	0.14	0.33	0.11
Quartile 3	0.25	0.34	0.22	0.39	0.68	0.25
Max	0.56	0.89	0.38	0.94	1.14	0.8
Mean	0.19 (*p* < 0.05)	0.29 (*p* < 0.05)	0.19 (NS)	0.3 (NS)	0.51 (NS)	0.19 (*p* < 0.05)

*LAS* laser alignment based on skin marking, *SGRT* surface guided radiation therapy, *NS* not significant

### Setup efficiency

Table 2 summarizes the initial setup and imaging times of the two setup methods. The average initial setup times were 185 s and 187 s using the LAS method and SGRT, respectively. No significant differences were observed in the initial setup time. However, the imaging time was reduced by approximately 13% using SGRT, which was statistically significant. The average total setup times considering both the initial setup time and imaging time were 314 s and 331 s with and without SGRT, respectively. It was confirmed that the efficiency of the total setup time in the SGRT was significantly improved by approximately 5% compared to that of the LAS method.

**Table 2:** Comparison of patient setup time with and without SGRT

	Initial setup (s)	Imaging (s)		Initial setup (s)	Imaging (s)		Total setup time (s)	
							LAS	SGRT
LAS			SGRT					
Min	121	94	Min	121	86	Min	215	224
Median	183	144	Median	190	125	Median	330	323
Quartile 1	169	121	Quartile 1	158	111	Quartile 1	301	284
Quartile 3	200	166	Quartile 3	213	137	Quartile 3	352	337
Max	271	94	Max	285	191	Max	446	437
Mean	185	146	Mean	187 (NS)	127 (*p* < 0.05)	Mean	331	314 (*p* < 0.05)

*LAS* laser alignment based on skin marking, *SGRT* surface guided radiation therapy, *NS* not significant

## Discussion

The accuracy and efficiency of using SGRT implemented on the recently installed Halcyon LINAC were evaluated based on both the phantom and patients. Many studies have reported the advantages of SGRT for various sites such as the head and neck, breast, abdomen, and pelvis [2–9, 14–22]. In particular, Fores-Martinez et al. reported that the use of SGRT on Halcyon allowed for a reduction of additional imaging during patient setup in non-SRS intracranial treatment, more accurate and faster initial setups for breast patients by reducing both re-alignment and repeat imaging [15]. Nguyen et al. [14] showed that the SGRT decrease the translational setup error significantly by up to 2.1 cm compared with tattoos and rotational error by approximately 35%. Most of these studies compared patient groups using two different methods, while this study compared the results of using both methods (LAS vs. SGRT) for one patient, showing that using SGRT can reduce total setup time as well as initial setup error. [1–9, 14–22]

To establish a reference for SGRT accuracy in measuring rotational error, using 3D printing, we fabricated an in-house breast phantom and a base plate capable of applying arbitrary rotational error. Unlike previous studies, because the inside of the phantom used in our study did not employ high-density material, auto rigid registration was mainly performed by matching the phantom surface, which had a relatively high contrast [14, 17]. Therefore, it was considered more suitable for evaluating the systematic accuracy of the rotational error for SGRT using only surface information. The maximum difference between the SGRT and auto rigid registration for pitch, roll, and yaw was 0.4° in our study, which is comparable to the results of a study by Mancosu et al., who reported an estimated maximum rotational error of ± 0.3° for SGRT [17].

Flores‐Martinez et al. [15] reported that there was a significant decrease in residual rotational error in pitch, but not in yaw and roll, for the patient setup in Halcyon with SGRT. By contrast, in our study, the average residual rotational error for roll was significantly decreased using SGRT. There was a slight decrease in the average residual rotational error in pitch, but it was not statistically significant, and yaw remained the same with and without the SGRT. Nguyen et al. reported that SGRT significantly reduced setup errors compared with tattoos in all translational directions in patients with breast cancer [14]. They also showed that additional information acquired by the SGRT system can improve the positioning accuracy of breast cancer patients compared with the laser-based setup [14]. However, our study showed that only the average residual translational error in the vertical direction decreased significantly, but increased in the longitudinal direction. The average longitudinal translational error was larger for both tattoos and SGRT in the study by Cravo et al. [18]. This errors are affected by respiratory induced chest wall motion, and a larger longitudinal difference was observed in our study. This is probably because the longitudinal direction, which is limited in accuracy by the flatness of the body surface, caused larger error in SGRT due to combination of respiratory induced motion [22].

Most studies have reported that SGRT offered a reduced average setup time compared with the tattoo or LAS methods [1–9]. However, SGRT increased the average initial setup time by approximately 2 s. It may take relatively more time to reach the initial setup because the initial setup should be performed within the tolerance for the quantitative value of 6-DOF. This allows therapists to use more information during the initial setup to create a more accurate patient setup. However, it was confirmed that the imaging time and the overall setup time were significantly reduced. Because the accuracy of the initial setup using SGRT was better, it could be shorter to match the reference image and daily kV CBCT image than LAS method. In particular, the imaging time can be reduced through the result of the better initial setup accuracy by reducing rotational error in Halcyon couch which is difficult to correct rotational error. Breast tissue, for which it is difficult to ensure setup reproducibility because it is soft and non-rigid, limits the accuracy of setups when using the tattoo or LAS methods. Sometimes, because PTV for breast cancer patient which include the supraclavicular lymph node is large and complex, SGRT which uses quantitative information of 6DOF is more suitable for breast cancer patient. In addition, as the 6-DOF information shown by the SGRT system can reduce inter-therapist variation, the reproducibility of the patient setup can be improved.

When SGRT was applied to the treatment of breast cancer patients in the Halcyon system, which has a limitation in correcting the rotational error, similarly to the other studies, our results showed its advantage in terms of accuracy and efficiency of patient setup. In addition, many studies have emphasized SGRT as a convenient and effective tool for initial patient setup and comfort because tattoos or markings were not applied to the patient's skin [3, 21]. Compared to the LAS method, patients were satisfied with the absence of marking within the skin; in particular, the additional task of therapists could be reduced because they did not need to reapply the skin marking. However, it is currently impossible for an SGRT system installed in Halcyon to monitor patient motion in the treatment isocenter located in the bore during treatment. Therefore, monitoring using an in-bore camera, which enables intra-fractional motion monitoring using the treatment camera, will be performed in a future study.

## Conclusion

The use of SGRT was confirmed to be valuable for reducing the daily setup error compared to the patient’s daily setup using ink-based skin marking. In SGRT, a more accurate patient setup can be effected by reducing the rotational errors that are difficult to correct owing to the lack of capacity for couch movement in Halcyon systems. In addition, it was confirmed that the imaging time could be reduced by using SGRT, and that treatment efficiency increased owing to a reduction in the total setup time. SGRT for breast cancer patients using Halcyon systems can facilitate efficient and accurate setups compared with the conventional ink-marking method.

## Data Availability

The datasets supporting the study conclusions are included within this manuscript.

## Abbreviations

SGRT: Surface-guided radiation therapy
3D: Three-dimensional
6-DOF: Six degrees of freedom
LAS: Laser alignment based on skin marking
WBRT: Whole breast radiotherapy
APBI: Accelerated partial breast irradiation
IMRT: Intensity-modulated radiation therapy
FFF: Flattening filter free
MLC: Multi-leaf collimator
kV CBCT: KV cone beam CT
VMAT: Volumetric modulated arc therapy
R&V: Record and verification
